# Crystal structure validation of verinurad *via* proton-detected ultra-fast MAS NMR and machine learning†

**DOI:** 10.1039/d4fd00076e

**Published:** 2024-07-17

**Authors:** Daria Torodii, Jacob B. Holmes, Pinelopi Moutzouri, Sten O. Nilsson Lill, Manuel Cordova, Arthur C. Pinon, Kristof Grohe, Sebastian Wegner, Okky Dwichandra Putra, Stefan Norberg, Anette Welinder, Staffan Schantz, Lyndon Emsley

**Affiliations:** a Institut des Sciences et Ingénierie Chimiques, École Polytechnique Fédérale de Lausanne (EPFL) CH-1015 Lausanne Switzerland lyndon.emsley@epfl.ch; b National Centre for Computational Design and Discovery of Novel Materials MARVEL, École Polytechnique Fédérale de Lausanne (EPFL) Lausanne Switzerland; c Data Science & Modelling, Pharmaceutical Sciences, R&D, AstraZeneca 43183 Gothenburg Sweden; d Swedish NMR Center, Department of Chemistry and Molecular Biology, University of Gothenburg 41390 Gothenburg Sweden; e Bruker BioSpin GmbH & Co KG 76275 Ettlingen Germany; f Early Product Development and Manufacturing, Pharmaceutical Sciences, R&D, AstraZeneca 43183 Gothenburg Sweden; g Oral Product Development, Pharmaceutical Technology & Development, Operations, AstraZeneca 43183 Gothenburg Sweden

## Abstract

The recent development of ultra-fast magic-angle spinning (MAS) (>100 kHz) provides new opportunities for structural characterization in solids. Here, we use NMR crystallography to validate the structure of verinurad, a microcrystalline active pharmaceutical ingredient. To do this, we take advantage of ^1^H resolution improvement at ultra-fast MAS and use solely ^1^H-detected experiments and machine learning methods to assign all the experimental proton and carbon chemical shifts. This framework provides a new tool for elucidating chemical information from crystalline samples with limited sample volume and yields remarkably faster acquisition times compared to ^13^C-detected experiments, without the need to employ dynamic nuclear polarization.

## Abstract 2

The structure of an active pharmaceutical ingredient (API) is validated using solely ultra-fast ^1^H-detected experiments and machine learning without the need for dynamic nuclear polarization.

## Graphical abstract

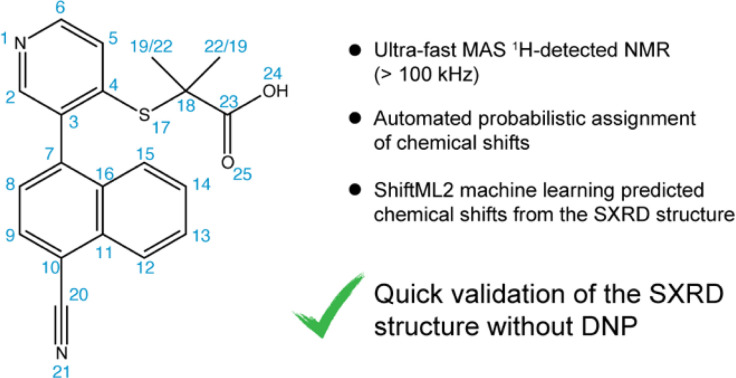

## Introduction

It is impossible to imagine any new drug development pipeline nowadays without a rigorous structure characterization step. This is partly due to the need for structure–activity relationships, partly due to concerns around stability and/or polymorphism, and partly due to regulatory pressure. Many active pharmaceutical ingredients (APIs) and drug formulations are powdered solids in their final form, so structural investigation in the native state requires well-established solid-state techniques capable of providing atomic-level resolution.

To this end, single-crystal X-ray diffraction (SXRD) is the method of choice.^1–7^ However, in the case of powders, SXRD is not appropriate, and powder X-ray diffraction (PXRD),^8–10^ electron diffraction^11–16^ and/or solid-state NMR spectroscopy^17–30^ are typically used instead. However, PXRD may suffer from peak broadening and spectral overlap due to low crystallinity,^31^ to the extent that the structure cannot be determined. Consequently, NMR can be used either to determine the crystal structure *de novo*,^32–37^ or to refine^38–45^ or validate^46–48^ a structure determined by X-ray diffraction methods.

The applicability of NMR crystallography to solve structures at the atomic level originates from the fact that the chemical shift is highly sensitive to the local atomic environment and can therefore report on the neighboring atoms and their spatial distribution.^26,33,37,46,49–55^ Using NMR crystallography, it is possible to investigate a broad range of solids extending from small organic molecules,^32,33,37^ to biomolecules,^56,57^ inorganic materials^58–66^ and even amorphous drugs.^49,50^ Atomic-level structures have been determined *de novo* from NMR chemical shifts in crystalline compounds for molecular solids,^32–34,37^ enzyme active sites,^41,67,68^ photovoltaic materials,^59,60,63^ and cementitious materials.^61,64,66^ NMR crystallography is extensively used not only to validate or determine the structure, but also to distinguish between multiple polymorphs of the same molecule.^69–77^

In chemical-shift-based NMR crystallography, the experimentally determined chemical shifts are compared to those calculated for (a set of) candidate crystal structures, and the candidate structure(s) with the lowest root-mean-square chemical-shift deviation (RMSD) is (are) then determined to be the true experimental structure. Confidence in the structure and positional uncertainties can then be determined using statistical methods.^78,79^ For validation, the candidate structure would typically have been determined by SXRD. For *de novo* determination, candidate structures are typically generated using crystal structure prediction (CSP)^80–84^ or molecular dynamics (MD)^85,86^ with a subsequent selection step of candidates having the lowest predicted lattice energy. For each of the candidates, chemical shifts are computed using density functional theory (DFT) methods, such as plane-wave gauge-including projector augmented wave (GIPAW) methods.^87–89^

Much progress has been made in solid-state NMR in the last 80 years that aims not only at extending its applications, but also at speeding up the NMR crystallography workflow. Tremendous advances have been achieved using machine-learning approaches that provide rapid chemical-shift predictions for molecular solids with DFT accuracy (dubbed ShiftML and ShiftML2).^90,91^ By combining ShiftML with the Cambridge Structural Database of three-dimensional structures, Cordova *et al.* developed a method for automated probabilistic assignment of the experimental chemical shifts for organic solids directly from their two-dimensional molecular structures.^92^ Due to hardware advances, we are now able to reach magic-angle spinning (MAS) rates of above 100 kHz. In combination with ^1^H resolution improvement techniques,^93–98^ ultra-fast MAS results in sufficient ^1^H line narrowing^99,100^ for the acquisition of high-resolution 1D and 2D ^1^H-detected spectra for typical molecular solids. For such materials, transitioning to ^1^H detection instead of ^13^C detection translates to faster acquisition due to the high sensitivity of protons, and no need for costly isotopic labelling or for hyperpolarization by dynamic nuclear polarization (DNP), which remain inaccessible for many NMR platforms around the world.

**Fig. 1:**
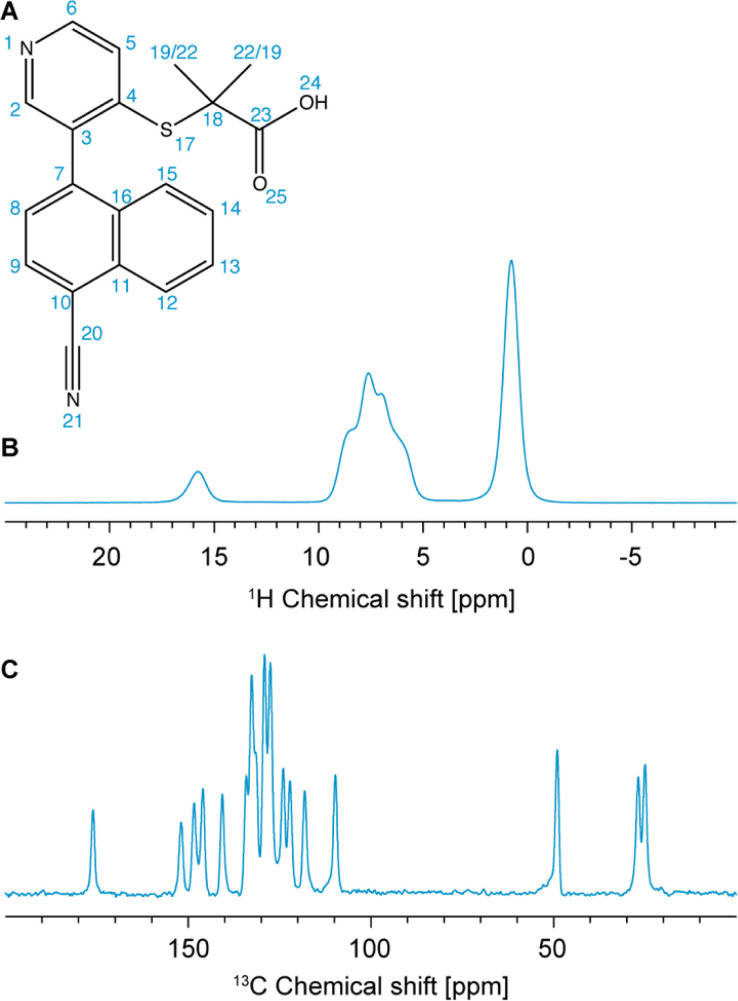
(A) Chemical structure of verinurad and labels corresponding to each atomic site. (B) 1D ^1^H spectrum at 160 kHz MAS. (C) ^13^C CP spectrum at 100 kHz MAS.

Here we demonstrate an NMR chemical-shift-led approach based on ^1^H detection at fast-MAS, in combination with the probabilistic assignment method, and machine-learned chemical shifts, to validate crystal structures. The method is illustrated with the validation of the structure of verinurad (RDEA3170) shown in Fig. 1A, a powerful inhibitor of the URAT1 uric acid transporter with potential pharmaceutical applications against hyperuricemia and gout.^101,102^

Previously, 18 solvates were found by a polymorph screen, of which anhydrous form A is the most stable.^103^ The latter exists as a racemic mixture of atropisomers due to restricted rotation about the cyano-naphthalene and pyridine rings (Fig. 1A). The crystal structure of form A was determined using SXRD, and it was found that it belongs to the *P*2_1_/*n* centrosymmetric space group with one molecule in the asymmetric unit.^103^ Importantly, for the assignment of the experimental chemical shifts, we rely solely on the ^1^H-detected 1D spectrum, and ^1^H-detected 2D hCH and ^1^H–^1^H DQ/SQ experiments at 160 kHz MAS, and on the probabilistic assignment of ^1^H and ^13^C shifts. The acquisition time needed for these experiments is roughly three times shorter than that needed to acquire a ^13^C DNP-refocused INADEQUATE. The information contained in the INADEQUATE spectrum that we would otherwise need to guide the assignment is replaced by the probabilistic assignment, which is sufficient to unambiguously assign 18 out of 20 ^13^C chemical shifts and 10 out of 12 ^1^H chemical shifts. The remaining carbons and protons corresponding to the two methyl groups have very similar local bonding environments, which makes their corresponding statistical distributions of chemical shifts very similar. Therefore, they are left ambiguously assigned. The experimental shifts are then compared to those predicted for the SXRD structure using ShiftML2 to validate the crystal structure. We find that the measured chemical shifts are in good agreement with calculated shifts from the SXRD structure.

## Experimental section

### Materials

Verinurad (2-[3-(4-cyanonaphthalen-1-yl)pyridin-4-yl]sulfanyl-2-methylpropanoic acid) was purchased from Selleckchem and was used without further recrystallization.

### NMR experiments

^1^H 1D, long-range hCH and ^1^H 2D DQ/SQ BABA spectra were acquired at 160 kHz MAS on a 18.8 T Bruker Avance Neo spectrometer corresponding to a ^1^H frequency of 800 MHz, using a Bruker 0.4 mm HCN CP-MAS probe. The sample temperature was regulated to 295 K using VT flow at 280 K.

The ^1^H-detected 2D long-range hCH and the ^13^C cross polarization (CP)^104,105^ at 100 kHz MAS and the VMAS ^1^H 1D dataset spectra for PIPNet at 40–100 kHz MAS were acquired on a Bruker Avance Neo spectrometer operating at 21.14 T (^1^H and ^13^C frequency of 900 MHz and 225 MHz, respectively) equipped with a 0.7 mm room temperature HCN CP-MAS probe. All the spectra at 100 kHz MAS were acquired at a constant VT temperature of 280 K to compensate for the frictional heating, resulting in a sample temperature of 295 K.

The one-dimensional ^1^H spectrum was recorded using a rotor-synchronized spin echo for background suppression. The echo delay was equal to two rotor periods. 2D short-range and long-range hCH^106^ NMR spectra were recorded with 250 us and 4 ms contact times, respectively, for the direct ^1^H–^13^C CP and 125 us and 2 ms contact times, respectively, for the back CP. WALTZ-16 (ref. 107) decoupling at a ^1^H and ^13^C nutation frequency of 10 kHz was applied during *t*_1_ and *t*_2_, respectively. In the short-range hCH, 512 increments of 200 scans each with a recycle delay of 1.5 s were acquired, resulting in 10.2 ms of evolution in the direct dimension and 5.12 ms of evolution in the indirect dimension. In the long-range hCH, 1024 increments of 144 scans each with a 1.5 s recycle delay were acquired. In the ^1^H 2D DQ/SQ BABA^108^ spectrum, one DQ excitation and reconversion period of one rotor period each was used. In total, 512 increments with 64 scans each were acquired with a repetition delay of 1.5 s. The acquisition times were 22.5 ms and 6.4 ms in *t*_2_ and *t*_1_, respectively.

States-TPPI is used for quadrature detection in the indirect dimension for all 2D experiments. In the 2D hCH spectra, 100 Hz exponential line broadening was used in the direct dimension.

Proton chemical shifts were referenced externally with respect to the adamantane CH_2_ group at 1.87 ppm. Carbon chemical shifts were referenced externally with respect to the adamantane CH group at 38.48 ppm.^109^

All the acquisition parameters and raw NMR data are available as described in the ESI (see Table S1†).

### SXRD structure relaxation

The SXRD structure was taken from ref. 103. The proton positions of the SXRD structure were optimized using the plane-wave DFT software Quantum ESPRESSO version 6.5 in order to correct any systematic error in the X-ray determinations of proton positions. The constrained optimizations were performed at the PBE level of theory using Grimme D3 dispersion correction and projector augmented wave scalar relativistic pseudopotentials with GIPAW reconstruction, S.pbe-nl-kjpaw_psl.1.0.0.UPF, H.pbe-kjpaw_psl.1.0.0.UPF, O.pbe-nl-kjpaw_psl.1.0.0.UPF, C.pbe-n-kjpaw_psl.1.0.0.UPF, N.pbe-n-kjpaw_psl.1.0.0.UPF. The wavefunction and charge density energy cutoffs were set to 120 and 960 Ry, respectively, and the relaxations were carried out without *k*-points.

### Chemical-shift predictions

Chemical shieldings were predicted for all ^1^H and ^13^C atomic sites using ShiftML2.^90,91^ In the training set of ShiftML2, all the training structures were relaxed using DFT-fixed cell geometry optimizations using the Quantum ESPRESSO (QE) electronic structure package with the PBE density functional, a Grimme D2 dispersion correction, wavefunction and charge density energy cut-offs of 60 and 240 Ry, respectively, and ultrasoft pseudopotentials with GIPAW reconstruction. The GIPAW NMR calculations were performed using the QE code with the same DFT parameters as for the structure relaxation, but using refined plane wave and charge density energy cut-offs of 100 and 400 Ry, respectively, a Monkhorst–Pack *k*-point grid with a maximum spacing of 0.06 Å^−1^, and the ultrasoft pseudopotentials with GIPAW reconstruction from the USSP pseudopotential database v1.0.0. Further details are given in ref. 90. The shieldings were converted to shifts using offsets of 30.78 and 170.04 ppm for ^1^H and ^13^C, respectively. Chemical shifts were also calculated using DFT (Tables S3 and S4 and other details are given in the ESI†).

## Results and discussion

### Assignment of NMR spectra

**Fig. 2:**
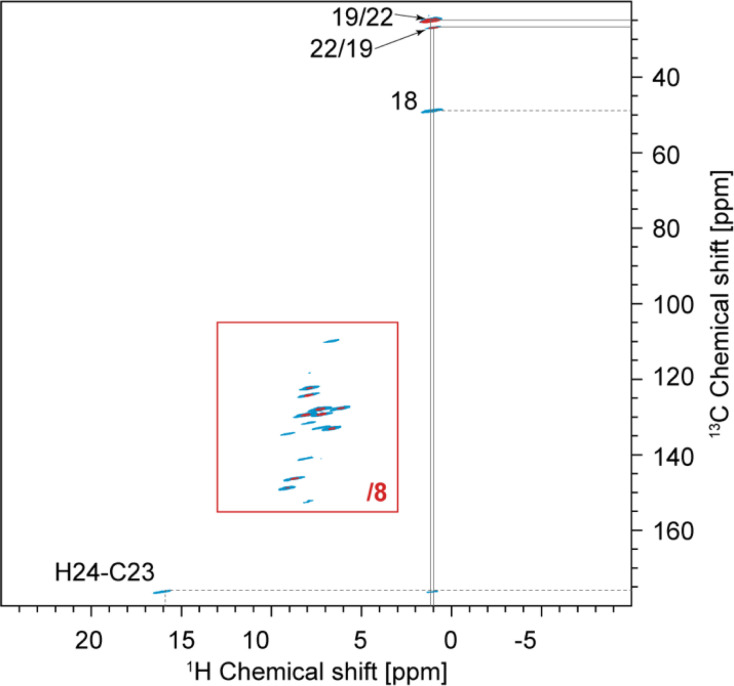
Complete 2D hCH long-range spectrum in blue (4 ms direct CP contact time) acquired at 160 kHz MAS overlaid with 2D hCH short-range spectrum in red (250 μs direct CP contact time) acquired at 100 kHz MAS. In the red spectrum, the contour levels were decreased by a factor 8 in the (13–3; 155–105) ppm region. This region is also given separately in Fig. 5. The label(s) corresponding to each assigned cross-peak are indicated on the spectrum. The solid lines are used for the protonated carbons and the dashed lines for the quaternary ones.

The one-dimensional ^1^H and ^13^C spectra are shown in Fig. 1B and C. The short- and long-range ^1^H–^13^C hCH correlation spectra are shown in Fig. 2. The methyl groups C19 and C22 are immediately recognized due to their ^1^H and ^13^C chemical shifts, typical for methyl groups (1.01 and 1.12 ppm for ^1^H, 27.0 and 25.1 ppm for ^13^C). The quaternary carbon C18 was assigned to 49.1 ppm due to its correlation with methyl groups in the long-range hCH spectrum (Fig. 2).

Similarly, H24 was unambiguously assigned at 15.91 ppm due to its characteristic chemical shift, and because it does not show any correlations in the short-range hCH (Fig. 2) and shows only one correlation with a carbon at 175.9 ppm in the long-range hCH (Fig. 2), which was then assigned to C23.

The assignment of the remaining seven quaternary carbons and nine CH groups, all of which are aromatic and resonate in the range from 100 to 160 ppm in the ^13^C dimension and 6 to 9 ppm in the ^1^H dimension, with linewidths of roughly 1 ppm for ^13^C and 0.5 ppm for ^1^H, requires more advanced methods.

**Fig. 3:**
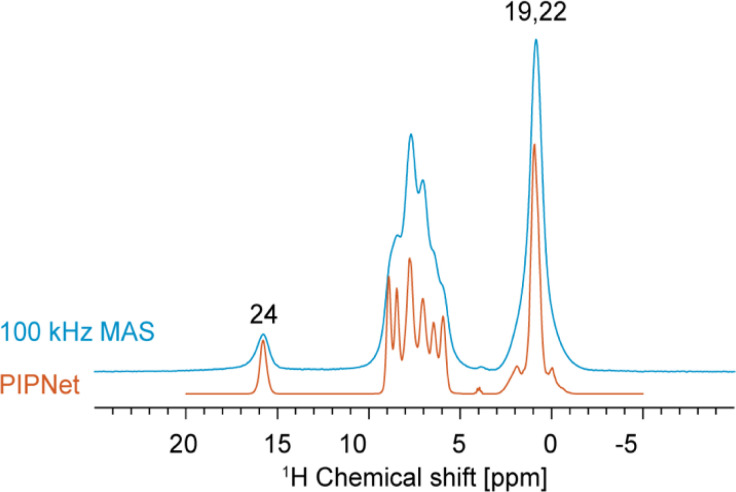
1D ^1^H spectrum acquired at 100 kHz MAS in blue overlaid with the PIPNet-predicted isotropic 1D spectrum in orange, obtained from 40 to 100 kHz MAS 1D ^1^H spectra.

First, to maximize ^1^H resolution, the 1D ^1^H spectrum of verinurad was acquired at variable MAS rates between 40 and 100 kHz MAS and this dataset was then used as input to predict the pure isotropic spectrum (free of any residual dipolar coupling) using the PIPNet approach.^93^ The pure isotropic spectrum is shown in Fig. 3, in comparison to the 100 kHz MAS spectrum, where we see a very significant improvement in resolution. Interestingly, the PIPNet prediction for the carboxylic proton showed significant line narrowing, proving that this ^1^H site is subject to significant residual dipolar coupling even at 100 kHz MAS.

**Fig. 4:**
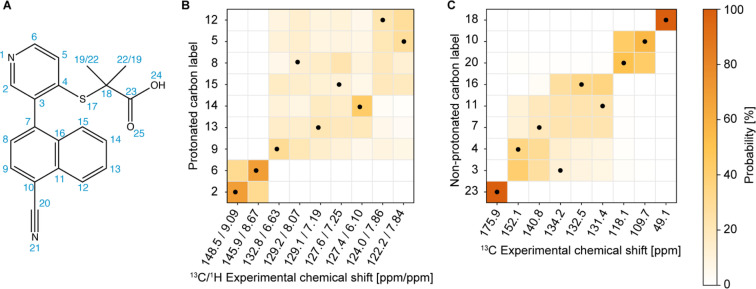
(A) Chemical structure of verinurad and labels corresponding to each atomic site. (B) and (C) Marginal individual assignment probabilities of the ^13^C (and ^1^H) chemical shifts after Bayesian inference of the global assignments of the (B) aromatic CH groups and (C) quaternary carbons. The dots indicate the experimentally determined correct assignment.

The next step in the process is to apply the Bayesian probabilistic assignment method.^92^ This approach uses a database of chemical shifts predicted using the ShiftML2 machine-learnt model for over 338 000 structures of molecular solids contained in the Cambridge Structural Database. For each nucleus in the molecule, a predicted distribution of chemical shifts is constructed from all the similar local molecular fragments present in the database. The ensemble of individual distributions for all the atoms in the molecule is then compared with the experimental peak lists to determine the most probable complete assignment. Details are given in ref. 92. Using the publicly available scripts at https://github.com/manucordova/ProbAsn, we obtained the results shown in Fig. 4.

**Fig. 5:**
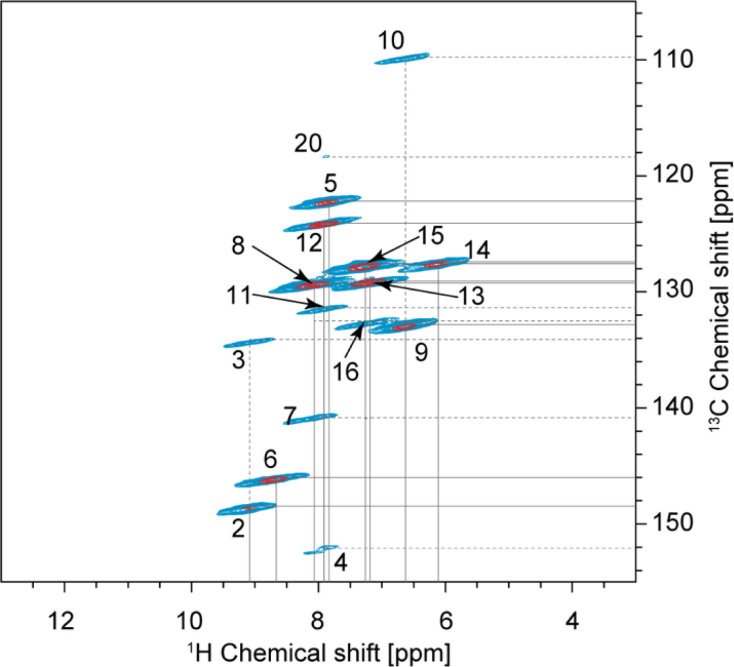
The aromatic region of the 2D hCH long-range spectrum in blue (4 ms direct CP contact time) at 160 kHz MAS overlaid with 2D hCH short-range spectrum in red (250 μs direct CP contact time) at 100 kHz MAS. The label(s) corresponding to each assigned cross-peak are indicated on the spectrum. The solid lines are used for the protonated carbons and the dashed lines for the quaternary ones.

Based on the probabilistic assignment of CH groups simultaneously using ^13^C and ^1^H experimental shifts from short-range hCH, C9 and H9 are assigned at 132.8 ppm and 6.63 ppm, respectively. From the long-range hCH (Fig. 5), H9 has a correlation with a quaternary carbon at 109.7 ppm, which is therefore assigned to C10. C20 is then assigned at 118.1 ppm, based on the probabilistic assignment of the quaternary carbons. Using the BABA spectrum (Fig. 7), it is found that C9 has a correlation with a proton at 8.07 ppm, which is therefore assigned to H8 attached to C8 at 129.2 ppm.

From the probabilistic assignment of CH groups, C14 and H14 are confidently assigned at 127.4 and 6.10 ppm, respectively (43% from the probabilistic assignment, compared to 17% for the next most probable assignment).

According to the probabilistic assignment, (H2; C2) and (H6; C6) are assigned to (9.09; 148.5) ppm and (8.67; 145.9) ppm, though with some ambiguity between them. Then, in the long-range hCH, the proton at 9.09 ppm has a correlation with a quaternary carbon at 134.2 ppm, whereas the proton at 8.67 ppm does not have any correlations with quaternary carbons. H2 and C2 are therefore confidently assigned at 9.09 ppm and 148.5 ppm, and H6 and C6 are assigned at 8.67 ppm and 145.9 ppm, respectively, which is in agreement with the most probable assignment. The quaternary carbon at 134.2 ppm is therefore assigned to C3.

From long-range hCH, H8 is correlated with three quaternary carbons at 140.8, 134.2, and 132.5 ppm. These three quaternary carbons most likely correspond to C3, C7, and C16 because they are the closest to C8 in the molecular structure of verinurad. The ^13^C peak at 134.2 ppm was previously assigned to C3. The ^13^C peak at 140.8 ppm does not show any other long-range correlations in hCH, as opposed to that at 132.5 ppm. As a result, C7 is assigned to 140.8 ppm because it is the only quaternary carbon that is not connected directly to any CH groups except C8, and therefore is the least expected to form long-range hCH correlations. C16 is therefore assigned at 132.5 ppm, which is in agreement with the probabilistic assignment of the quaternary carbons. Consequently, having a long-range hCH correlation with C16, H15 is assigned at 7.25 ppm and C15 at 127.6 ppm.

Three CH groups remain unassigned at this point, C5, C12, and C13. Their ^13^C and ^1^H experimental shifts in the short-range hCH spectrum are (122.2; 7.84), (124.0; 7.86), and (129.1; 7.19) ppm. C13 is assigned at 129.1 ppm because it has very low probability to be at either 122.2 or 124.0 ppm according to the probabilistic assignment of the CH groups, whereas C5 and C12 have extremely low probability to be at 129.1 ppm.

C4 and C11 are assigned at 152.1 ppm and 131.4 ppm, respectively. The only hCH correlations for quaternary carbons left unassigned are 152.1 and 131.4 ppm, but based on the probabilistic assignment, C4 has extremely low probability to be at 131.4 ppm and *vice versa*.

C11 has a long-range hCH correlation with a proton at 7.86 ppm that is therefore assigned to H12, with C12 being assigned at 124.0 ppm. The last CH group left unassigned is C5–H5 which is therefore attributed the 13C and 1H chemical shifts of 122.2 and 7.84 ppm, respectively. Such an assignment of C5 and C12 protonated aromatic groups is in agreement with the ShiftML2 predicted shifts of 122.0 and 123.5 ppm for C5 and C12, respectively, which are very close to the experimental values.

### Validation of the SXRD structure

**Fig. 6:**
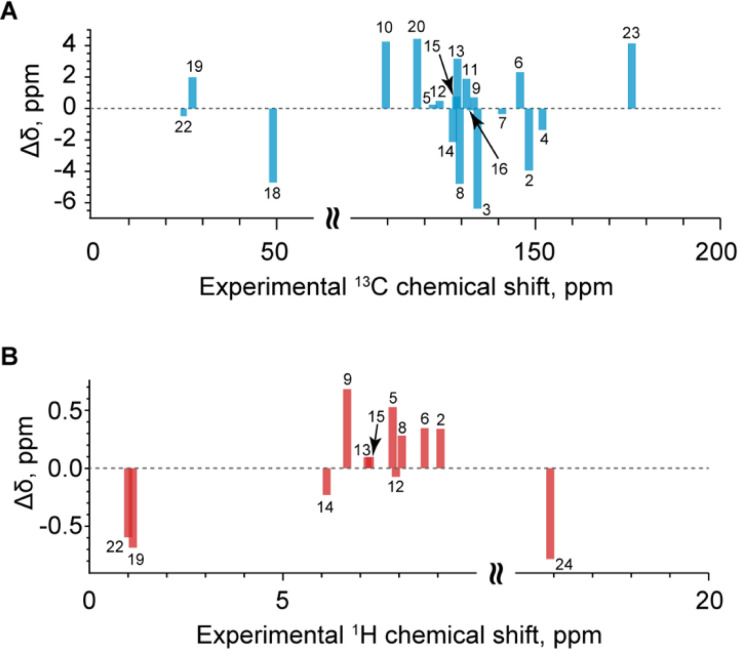
(A) Difference between the experimental and predicted ^13^C chemical shifts Δ*δ* per atomic site as a function of experimental ^13^C chemical shift. (B) Difference between the experimental and predicted ^1^H chemical shifts Δ*δ* per atomic site as a function of experimental ^1^H chemical shift. The horizontal gray dashed lines in (A) and (B) represent Δ*δ* = 0 ppm.

With the assigned chemical shifts in hand, we can now compare the measured chemical shifts with those predicted by ShiftML2 for the crystal structure obtained *via* SXRD with DFT-relaxed proton positions. Fig. 6 shows the comparison between the measured and predicted shifts. The RMSD between experiment and calculation is 3.1 ppm for ^13^C and 0.46 ppm for ^1^H. These deviations are within the expected errors for the ShiftML2 method used here, which have been estimated at 4.53 ppm for ^13^C and 0.47 ppm for ^1^H.^90^ When looking in detail at the correlation between experiment and calculation, there are no significant outliers. We conclude that the NMR results validate the single-crystal X-ray structure.

### Intermolecular correlations observed in the BABA spectrum

In the 2D DQ/SQ BABA experiment, the correlations originate from dipolar coupling between nearby protons instead of scalar couplings. As a result, intermolecular correlations can be observed in BABA spectra if the distance between the coupled spins is small enough.

**Fig. 7:**
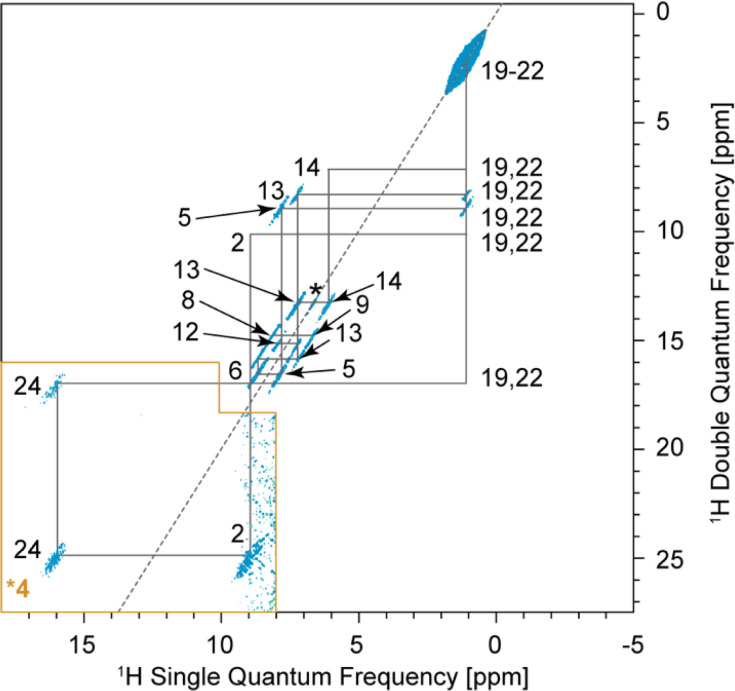
2D ^1^H–^1^H BABA spectrum of verinurad obtained at 160 kHz MAS. The contour levels were increased by a factor 4 in the (18–8; 27.5–16) ppm region. The dotted grey line indicates the spectral 2 : 1 “diagonal”. The solid grey lines indicate the connectivities between cross peaks. The asterisk indicates the intermolecular correlation between equivalent H9 atomic sites separated by 2.55 Å.

In the BABA spectrum of verinurad (Fig. 7), H2 has a correlation with H24, pointing towards an intermolecular H-bond of which H24 is the donor and N1 is the acceptor. H9 presents an autocorrelation peak that originates from the intermolecular contact between equivalent H9 atomic sites separated by 2.55 Å in the SXRD structure where the proton positions were relaxed *via* DFT. H6 shows a BABA correlation with either H13 or H15, but given the small chemical-shift difference between H13 and H15 of only 0.06 ppm, this correlation cannot be assigned further. However, from the SXRD structure, this correlation is confidently assigned to H13 where the intermolecular distance from H6 is 2.4 Å and the smallest distance between H6 and H15 is 5.1 Å. For chemical-shift-driven structure prediction, such intermolecular BABA correlations can serve as constraints when no XRD structure is available.

As for the chemical shifts, we conclude that all the observed inter-molecular correlations are in line with expectations from the SXRD structure.

## Conclusions

We have demonstrated an NMR chemical-shift-led approach based on ^1^H detection at fast-MAS, in combination with the probabilistic assignment method, and machine-learned chemical shifts, to validate the crystal structure of verinurad.

The assignment of the experimental chemical shifts relied solely on the ^1^H-detected experiments at 160 kHz MAS, together with the probabilistic assignment of ^1^H and ^13^C shifts. The chemical shifts predicted for the SXRD structure using ShiftML2 are found to be the same, to within error, as those measured experimentally, thereby validating the SXRD structure.

The ^1^H-based assignment approach outlined here should be applicable to crystal structure validation or refinement for other small organic molecule APIs and is faster than the current standard NMR crystallography methodology.

## Author contributions

Manuel Cordova: software (lead); investigation (equal); review and editing (equal). Lyndon Emsley: conceptualization (equal); project administration (lead); formal analysis (equal); supervision; writing – original draft (supporting); writing – review and editing (lead). Kristof Grohe: investigation (supporting); resources; writing – review and editing (equal). Jacob B. Holmes: formal analysis (equal); investigation (equal); software; visualization; writing – review and editing (equal). Sten Nilsson Lill: software; writing – review and editing (equal). Pinelopi Moutzouri: formal analysis (equal); investigation (equal); validation; writing – review and editing (equal). Stefan Norberg: supervision; writing – review and editing (equal). Arthur C. Pinon: formal analysis (equal); investigation (equal); validation; writing – review and editing (equal). Okky Dwichandra Putra: supervision; writing – review and editing (equal). Staffan Schantz: conceptualization (equal); supervision; writing – review and editing (equal). Daria Torodii: data curation; formal analysis; investigation (equal); visualization (lead); writing – original draft preparation (lead); writing – review and editing (equal). Sebastian Wegner: investigation (supporting); resources; writing – review and editing (equal). Anette Welinder: supervision; writing – review and editing (equal).

## Conflicts of interest

There are no conflicts to declare. AstraZeneca authors will disclose that they are employees of AstraZeneca and that they have ownership, options, or interests in AstraZeneca stock.

## Supplementary Material

FD-255-D4FD00076E-s001
