# Supplementary material for: Crystal structure validation of verinurad via proton-detected ultra-fast MAS NMR and machine learning
Source: Faraday Discuss. 2024 Jul 17;255:143–58. doi: 10.1039/d4fd00076e (PMC11411500; doi:10.1039/d4fd00076e)
Supplement: FD-255-D4FD00076E-s001 [file FD-255-D4FD00076E-s001.pdf]

Supplementary Information (SI) for Faraday Discussions.
This journal is © The Royal Society of Chemistry 2024

Crystal structure validation of verinurad via proton-detected ultra-fast MAS NMR and machine learning

Daria Torodii[1], Jacob B. Holmes[1,2], Pinelopi Moutzouri[1], Sten Nilsson Lill[3], Manuel Cordova[1,2], Arthur C. Pinon[4], Kristof Grohe[5], Sebastian Wegner[5], Okky Dwichandra Putra[6], Stefan Norberg[7], Anette Welinder[7], Staffan Schantz[7], Lyndon Emsley[1,2]*

[1]Institut des Sciences et Ingénierie Chimiques, École Polytechnique Fédérale de Lausanne (EPFL), CH-1015 Lausanne, Switzerland

[2]National Centre for Computational Design and Discovery of Novel Materials MARVEL, École Polytechnique Fédérale de Lausanne (EPFL), Lausanne, Switzerland

[3]Data Science & Modelling, Pharmaceutical Sciences, R&D, AstraZeneca, 43183 Gothenburg, Sweden

[4]Swedish NMR Center, Department of Chemistry and Molecular Biology, University of Gothenburg, 41390 Gothenburg, Sweden

[5]Bruker BioSpin GmbH & Co KG, 76275 Ettlingen, Germany

[6]Early Product Development and Manufacturing, Pharmaceutical Sciences, R&D, AstraZeneca, 43183 Gothenburg, Sweden

[7]Oral Product Development, Pharmaceutical Technology & Development, Operations, AstraZeneca, 43183 Gothenburg, Sweden

Supporting Information

Raw data statement: All data and code used are available from https://doi.org/10.24435/materialscloud:qk-x9 under the license CC-BY-4.0 (Creative Commons Attribution-ShareAlike 4.0 International).

Table of Contents

1. Experimental Details 3
2. Assignment 4
3. Probabilistic assignment 4
4. Chemical shift calculations 5
5. References 6

1. Experimental Details

All the raw data and the associated pulse sequences and full acquisition parameters are available at: https://doi.org/10.24435/materialscloud:qk-x9.

The sample was purchased from Selleckchem, and the as received sample was packed in 0.7 and 0.4 mm rotors after being crushed with a mortar and pestle. All the experiments at 100 kHz MAS were acquired using a Bruker 0.7 mm room temperature HCN CP-MAS probe at a magnetic field of 21.1 T corresponding to a ^{1}H frequency of 900 MHz. The temperature was kept constant using a VT flow calibrated to compensate for the frictional heating measured externally using KBr. All the experiments at 160 kHz MAS were acquired on 18.8 T Bruker Avance Neo spectrometer corresponding to a ^{1}H frequency of 800 MHz using a Bruker 0.4 mm HCN CP-MAS probe. The sample temperature was regulated to 295 K using VT flow at 280 K. A States-TPPI acquisition scheme was used in all 2D experiments to obtain phase-sensitive two-dimensional spectra. All spectra were phase and baseline corrected. An exponential window function of 100 Hz in the direct dimension was applied prior to Fourier transformation of the hCH spectra. An exponential window function of 100 was applied to the 1D ^{1}H-^{13}C CP MAS spectrum prior to Fourier transformation of the hCH spectra. No window function was applied to the ^{1}H 1D MAS spectra.

For the PIPNet ^{1}H spectrum a series of 31 spectra were recorded at spinning rates of 40 to 100 kHz MAS, and then used as input into PIPNet as described in reference 1, yielding the spectrum shown in figure 3.

Table S1. NMR experimental details for verinurad.

Experiment	MAS rate (kHz)	VT (K)	^{1}H 90° RF amplitude (kHz)	CP contact time, ms	recycle delay (s)	Number of FID points	SW (kHz)	Size of real spectrum:	Number of scans
1D ^{1}H echo	160	280	250	-	1.5	2048	50	4096	64
hCH long-range	160	280	227	4 (direct CP) & 2 (back CP)	1.5	4096 (F2) / 1024 (F1)	200 (F2) / 44 (F1)	8192 (F2) / 2048 (F1)	144
hCH short-range	100	285	299	0.25 (direct CP) & 0.125 (back CP)	1.5	4096 (F2) / 512 (F1)	200 (F2) / 50 (F1)	8192 (F2) / 1024 (F1)	200
^{1}H-^{1}H BABA	160	280	250	-	1.5	4096 (F2) / 512 (F1)	91 (F2) / 40 (F1)	8192 (F2) / 1024 (F1)	64
^{13}C CP	100	280	303	2	1.5	4096	139	16384	8192

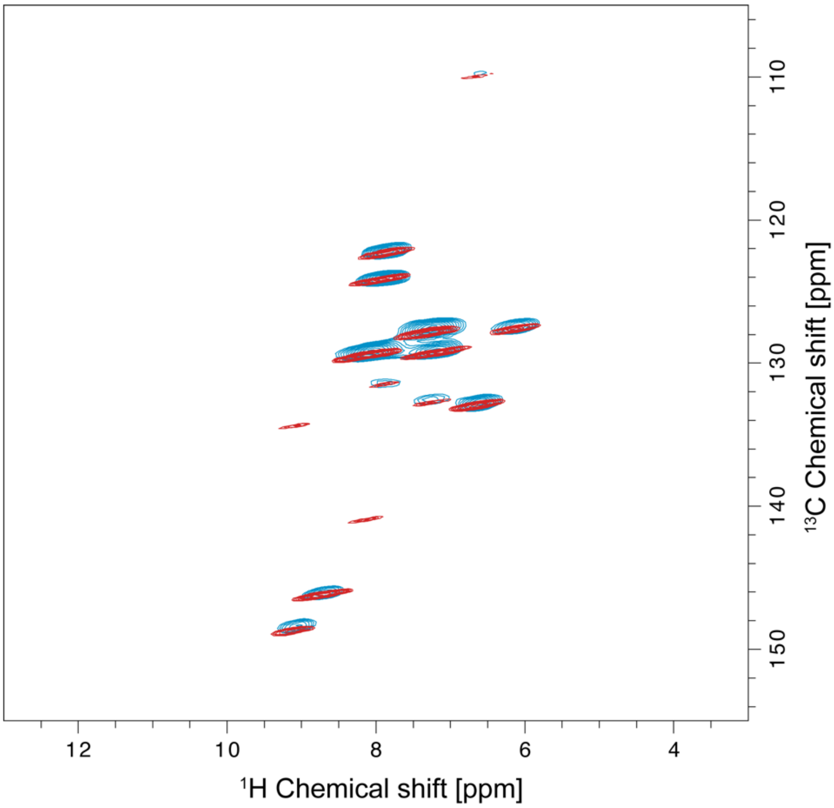

Figure S1. The aromatic region of the 2D hCH long-range spectrum of verinurad at 100 kHz MAS in blue (2 ms contact time) and at 160 kHz MAS in red (4 ms contact time).

2. Assignment

Table S2. Solid-state NMR assignment of ^{1}H and ^{13}C chemical shifts of verinurad as received.

Atom label	^{13}C Chemical Shift, ppm	^{1}H Chemical Shift, ppm
2	148.5	9.09
3	134.2	
4	152.1	
5	122.2	7.84
6	145.9	8.67
7	140.8	
8	129.2	8.07
9	132.8	6.63
10	109.7	
11	131.4	
12	124.0	7.86
13	129.1	7.19
14	127.4	6.10
15	127.6	7.25
16	132.5	
18	49.1	
19	25.1 / 27.0	1.12 / 1.01
20	118.1	
22	27.0 / 25.1	1.01 / 1.12
23	175.9	
24		15.91

3. Probabilistic assignment

The input files used for the Bayesian probabilistic assignment (verinurad_13c.in and verinurad_C-H_mult_1.in) and the complete output data generated are given as additional Supplementary Files.

4. Chemical shift calculations

The input files used for the DFT and ShiftML2 and the complete output data generated are given as additional Supplementary Files. The optimization of the proton positions of the SXRD structure and the NMR computations were carried out using the plane-wave DFT software Quantum ESPRESSO version 6.5 with the PBE density functional, a Grimme D3 dispersion correction, wavefunction and charge density energy cut-offs of 120 and 960 Ry, respectively, and ultrasoft pseudopotentials with GIPAW reconstruction. The GIPAW NMR calculations were performed using the QE code with the same DFT parameters as for the structure relaxation but using refined plane wave and charge density energy cut-offs of 120 and 960 Ry, respectively, a 4 x 2 x 1 Monkhorst–Pack k-point grid (with a maximum spacing of 0.05 $Å^{-1}$), and the ultrasoft pseudopotentials with GIPAW reconstruction from the USSP pseudopotential database v1.0.0. Conversion of σ_{calc} to δ_{calc} were performed using equation 1.

$$\delta_{calc} = \sigma_{ref} - b\sigma_{calc} \quad (1)$$

The rescaling parameters σ_{ref} and *b* were determined by linear regression between computed shielding and experimental shifts, permuting any ambiguities. The RMSE obtained from GIPAW NMR calculations are 0.30 ppm for ^{1}H and 2.48 ppm for ^{13}C.

Table S3. ^{1}H and ^{13}C ShiftML2 predicted chemical shifts of verinurad.

Atom label	^{13}C Chemical Shift, ppm	^{1}H Chemical Shift, ppm
2	152.4	8.75
3	140.6	
4	153.5	
5	122.0	7.32
6	143.6	8.32
7	141.2	
8	134.0	7.79
9	132.1	5.95
10	105.5	
11	129.5	
12	123.5	7.92
13	125.9	7.09
14	129.5	6.33
15	126.8	7.15
16	132.6	
18	53.8	
19	25.0	1.80
20	113.7	
22	25.6	1.61
23	171.8	
24		16.69

Table S4. ^{1}H and ^{13}C DFT calculated chemical shifts of verinurad.

Atom label	^{13}C Chemical Shift, ppm	^{1}H Chemical Shift, ppm
2	148.4	8.49
3	136.3	
4	155.9	
5	120.5	7.31
6	143.1	8.46
7	143.0	
8	128.4	8.12
9	134.3	7.08
10	108.0	
11	130.4	
12	124.8	8.05
13	126.7	7.38
14	127.3	6.35
15	128.9	7.21
16	132.4	
18	49.6	
19	27.2	1.30
20	123.5	
22	23.2	0.90
23	169.6	
24		16.08

5. References

(1) Cordova, M.; Moutzouri, P.; Simões de Almeida, B.; Torodii, D.; Emsley, L. Pure isotropic proton NMR spectra in solids using deep learning. *Angewandte Chemie International Edition* **2023**, *62* (8), e202216607.
